# Enhanced Effectiveness of Extracorporeal Shock Wave Lithotripsy for Residual Stones Post-percutaneous Nephrolithotomy: A Comparative Study With Primary Stones of Similar Size

**DOI:** 10.7759/cureus.92918

**Published:** 2025-09-22

**Authors:** Mushtaq Hussain, Ahmad Waleed, Muhammad Danial Iqbal, Kanwal Naz, Tanzeel Gazder, Syed Saeed Abidi

**Affiliations:** 1 Urology, Sindh Institute of Urology and Transplantation, Karachi, PAK; 2 Urology, Queen Elizabeth Hospital Birmingham, Birmingham, GBR; 3 Urology, Russell's Hall Hospital, Dudley, GBR

**Keywords:** extracorporeal shock wave lithotripsy, percutaneous nephrolithotomy, renal stones, residual stones, shock wave therapy

## Abstract

Introduction: Renal stones are a common health problem worldwide, with rising prevalence and high recurrence rates. Extracorporeal shock wave lithotripsy (ESWL) and percutaneous nephrolithotomy (PCNL) are established treatments. While ESWL is well studied for primary renal stones, its effectiveness for post-PCNL residual stones is less well defined.

Objective: This study aims to compare the stone-free rate of ESWL in patients with post-PCNL residual stones versus those with primary renal stones of the same size (5-15 mm).

Methods: This prospective cohort study was conducted at the Sindh Institute of Urology and Transplantation, Karachi, from January to July 2022. A total of 108 patients with solitary renal stones (5-15 mm) were enrolled into two equal groups: post-PCNL residual stones and primary stones. ESWL was performed using a Dornier Compact Sigma lithotripter, delivering 2,500-3,500 shocks per session at 60-90 shocks/minute, with energy titrated up to a maximum of 6 J according to patient tolerance. Stone-free status was defined as the absence of residual fragments >4 mm on ultrasonography and kidney-ureter-bladder X-ray at eight weeks.

Results: The mean age of the cohort was 35.09 ± 10.72 years, mean BMI 25.06 ± 3.33 kg/m², and mean stone size 1.16 ± 0.27 cm. Overall stone clearance was achieved in 78 patients (72.2%). Clearance was significantly higher in the post-PCNL group (48/54; 88.9%) compared with the primary stones group (30/54; 55.6%) (p < 0.01). Patients with post-PCNL stones were more likely to be stone-free (relative risk (RR) 1.60, 95% CI 1.24-2.07). No major complications were observed; minor flank pain and transient hematuria occurred in 12% of patients and resolved conservatively.

Conclusion: ESWL achieved significantly higher stone-free rates in post-PCNL residual stones compared with primary renal stones of the same size. Given its safety, accessibility, and non-invasive nature, ESWL should be considered a first-line treatment option for small residual fragments following PCNL.

## Introduction

Renal stone disease is a significant global health problem, with a prevalence ranging from 5% to 19% in industrialized countries and a rising incidence worldwide [1]. In Pakistan, the burden is particularly high due to climatic conditions, dietary patterns, and limited access to healthcare [2,3]. The disease has both health and economic consequences, with recurrence rates reaching up to 50% within 5-10 years [4].

Treatment strategies depend on stone size, location, composition, and patient characteristics. For stones up to 1.5 cm, extracorporeal shock wave lithotripsy (ESWL) is widely accepted as a first-line treatment because it is non-invasive. Reported success rates range from 13.6% to 91.2% [5-7], reflecting variability related to stone size, density, location, patient body mass index, and lithotripter technology. For larger or more complex stones (>2 cm), percutaneous nephrolithotomy (PCNL) remains the gold standard, with stone-free rates of 40-90% reported in different series [5,6].

A persistent challenge in stone management is the presence of residual fragments after PCNL, with rates reported between 10% and 60% [8]. Although fragments ≤4 mm are often labeled "clinically insignificant," some studies use thresholds of ≤2-3 mm, and even small residuals can enlarge, cause symptoms, or lead to recurrence [9-11]. Options for managing these fragments include repeat PCNL, flexible ureteroscopy, and ESWL [12]. While repeat PCNL offers high clearance rates, it requires general anesthesia, carries bleeding and infection risks, and prolongs hospital stay. ESWL, in contrast, is less invasive and can be performed on an outpatient basis.

Several studies suggest ESWL may be more effective for post-PCNL residual stones than for primary stones of the same size. El-Nahas et al. reported stone-free rates of 81.4% in post-PCNL residual stones compared with 59.7% in primary stones [13]. However, prospective cohort data directly comparing these groups remain limited. Addressing this gap is important to guide clinical decision-making, reduce unnecessary retreatments, and optimize healthcare resources [14].

The objective of this study was to compare the stone-free rate of ESWL in patients with post-PCNL residual stones and those with primary renal stones of equal size (5-15 mm), to guide optimal management in our setting.

## Materials and methods

This prospective cohort study was conducted in the Department of Urology at the Sindh Institute of Urology and Transplantation (SIUT), Karachi, from January 24, 2022, to July 23, 2022. The six-month recruitment window was selected to include consecutive eligible patients while ensuring consistent treatment protocols and resource availability. A total of 108 patients, aged 18-60 years, with solitary renal stones measuring 5-15 mm were enrolled after radiological confirmation by ultrasonography or kidney-ureter-bladder (KUB) X-ray.

Two equal groups were formed: the post-PCNL residual stones group, comprising patients with residual stones following percutaneous nephrolithotomy, and the primary stones group, comprising patients with stones of the same size who had not undergone prior PCNL. Eligible stones were located in the upper calyx, lower calyx, or pelviureteric junction (PUJ).

Exclusion criteria included multiple stones, active urinary tract infection, uncorrected coagulopathy, pregnancy, and anatomical abnormalities preventing ESWL. For the primary stone group, patients with stone density greater than 1000 Hounsfield units (HU) on CT were excluded, as this threshold is known to predict poor ESWL outcomes. However, in the post-PCNL residual stone group, patients were included regardless of their pre-PCNL HU value, since fragments may already have been structurally altered during PCNL and therefore not directly comparable to intact primary stones.

All patients underwent ESWL using a Dornier Compact Sigma lithotripter (Dornier MedTech, Germany). Each session involved 2,500-3,500 shocks delivered at a frequency of 60-90 shocks per minute. Energy was gradually increased from low settings to a maximum of 6 joules, tailored to patient tolerance and the degree of fragmentation observed on fluoroscopy or ultrasonography.

Patients were reviewed every two weeks after treatment and evaluated at eight weeks with KUB X-ray and ultrasonography to determine stone-free status. Stone-free status was defined as the absence of residual fragments larger than 4 mm, consistent with guideline recommendations and widely used definitions in the ESWL literature.

Data collected included age, gender, body mass index (BMI), stone size, stone location, and stone-free status. Statistical analysis was performed using IBM SPSS Statistics for Windows, Version 25 (Released 2018; IBM Corp., Armonk, New York). Quantitative variables such as age, BMI, and stone size were expressed as mean ± standard deviation, while qualitative variables were expressed as frequencies and percentages. Comparisons between groups were made using the chi-square test for categorical variables and the independent t-test for continuous variables. Stratification was carried out for age, gender, BMI, stone size, and stone location to assess effect modification, and a p-value ≤0.05 was considered statistically significant.

Ethical approval for the study was obtained from the Institutional Review Board of SIUT. Written informed consent was obtained from all participants prior to enrollment, and the study was conducted in accordance with the Declaration of Helsinki.

## Results

A total of 108 patients were analyzed, with 54 in the post-PCNL residual stones group and 54 in the primary stones group. The mean age of the overall cohort was 35.09 ± 10.72 years, with no significant difference between groups (p = 0.49). BMI and stone size were also comparable (p = 0.79 and p = 0.76, respectively). Males comprised 68.5% of the total sample, with a similar distribution between groups (p = 0.68).

Baseline characteristics, including stone location, are shown in Table 1. Lower calyceal stones were most frequent in both groups (37%), followed by middle calyceal (33.3%), renal pelvis (19.4%), upper calyceal (8.3%), and PUJ stones (1.9%). No statistically significant difference was found in stone location distribution (p > 0.05).

**Table 1:** Baseline characteristics and stone location Clearance is defined as the absence of residual fragments >4 mm on follow-up imaging at eight weeks. Test statistics are from chi-square tests unless otherwise stated. Fisher’s exact test is used when expected counts are <5. PCNL: percutaneous nephrolithotomy; BMI: body mass index; PUJ: pelviureteric junction.

Variable	Total (N = 108)	Post-PCNL residual stones (n = 54)	Primary stones (n = 54)	Test statistic	p-value
Age (years)	35.09 ± 10.72	34.33 ± 10.49	35.85 ± 10.99	t = -0.74	0.464
BMI (kg/m²)	25.06 ± 3.34	25.14 ± 3.40	24.98 ± 3.28	t = 0.25	0.804
Stone size (cm)	1.16 ± 0.27	1.17 ± 0.28	1.15 ± 0.27	t = 0.38	0.706
Male	74 (68.5%)	36 (66.7%)	38 (70.4%)	χ² = 0.043	0.836
Female	34 (31.5%)	18 (33.3%)	16 (29.6%)	—	—
Upper calyx	9 (8.3%)	3 (5.6%)	6 (11.1%)	Fisher’s exact	0.489
Middle calyx	36 (33.3%)	14 (25.9%)	22 (40.7%)	χ² = 2.042	0.153
Lower calyx	40 (37.0%)	21 (38.9%)	19 (35.2%)	χ² = 0.040	0.842
Renal pelvis	21 (19.4%)	14 (25.9%)	7 (13.0%)	χ² = 2.128	0.145
PUJ	2 (1.9%)	2 (3.7%)	0 (0.0%)	Fisher’s exact	0.495

With respect to stone density, patients with primary stones >1000 HU were excluded, while post-PCNL patients were included regardless of HU. Although exact subgroup numbers were not stratified by HU in this analysis, several post-PCNL patients had preoperative CT values exceeding 1000 HU and still achieved clearance following ESWL, suggesting that the PCNL process may weaken stone structure and enhance subsequent fragmentation.

Overall, complete stone clearance was achieved in 72.2% of patients. The clearance rate was significantly higher in the post-PCNL group (48/54; 88.9%) compared to the primary stones group (30/54; 55.6%) (p < 0.01). Patients with post-PCNL stones were 1.6 times more likely to be stone-free compared to those with primary stones (RR 1.60, 95% CI 1.24-2.07).

Subgroup clearance rates are summarized in Table 2, showing consistently higher rates in post-PCNL patients across most age, gender, BMI, and stone size categories. Exceptions included upper calyceal and PUJ stones, where clearance rates were similar between groups.

**Table 2:** Complete stone clearance rates by subgroup Clearance is defined as the absence of residual fragments >4 mm on follow-up imaging at eight weeks. Test statistics are from chi-square tests unless otherwise stated. Fisher’s exact test is used when expected counts are <5. PCNL: percutaneous nephrolithotomy; BMI: body mass index; PUJ: pelviureteric junction.

Subgroup	Post-PCNL residual stones, n (%)	Primary stones, n (%)	Test statistic	p-value
Overall clearance	48 (88.9%)	30 (55.6%)	χ² = 13.34	<0.001
Age 20–34 years	18 (90.0%)	21 (65.6%)	Fisher’s exact	0.057
Age 35–60 years	30 (88.2%)	9 (40.9%)	Fisher’s exact	<0.001
Male	31 (86.1%)	20 (52.6%)	χ² = 8.17	0.004
Female	17 (94.4%)	10 (62.5%)	Fisher’s exact	0.035
BMI ≤ 24.99 kg/m²	29 (90.6%)	14 (53.8%)	Fisher’s exact	0.002
BMI ≥ 25.00 kg/m²	19 (86.4%)	16 (57.1%)	Fisher’s exact	0.033
Stone 0.7–1.10 cm	27 (100%)	13 (61.9%)	Fisher’s exact	<0.001
Stone 1.11–1.50 cm	21 (77.8%)	17 (51.5%)	χ² = 3.35	0.067
Upper calyx	3 (100%)	6 (100%)	—	1
Middle calyx	19 (86.4%)	6 (42.9%)	Fisher’s exact	0.01
Lower calyx	18 (94.7%)	12 (57.1%)	Fisher’s exact	0.009
Renal pelvis	5 (71.4%)	8 (57.1%)	Fisher’s exact	0.656
PUJ	1 (50%)	0 (0.0%)	Fisher’s exact	1

Safety outcomes

No major complications occurred in either group. Minor flank pain and transient hematuria were observed in 12% of patients. All cases were resolved with conservative management.

## Discussion

This prospective cohort study demonstrated that ESWL is significantly more effective in achieving stone-free status in patients with post-PCNL residual stones compared to those with primary renal stones of the same size. The overall stone-free rate in our study was 72.2%, with a marked difference between the post-PCNL group (88.9%) and the primary stones group (55.6%). Patients with post-PCNL stones were 1.6 times more likely to achieve stone-free status (RR 1.60, 95% CI 1.24-2.07), underscoring the magnitude of benefit.

Our results align closely with El-Nahas et al. [13], who reported stone-free rates of 81.4% in post-PCNL residual stones compared to 59.7% in primary stones. Similarly, Auge et al. [15] and Aminsharifi et al. [16] found that ESWL achieves high clearance rates for residual fragments ≤15 mm. Compared with those studies, our cohort was younger and excluded stones >1000 HU, which may partly explain the slightly higher clearance rates. In addition, the prospective design, uniform imaging follow-up, and balanced group sizes strengthen our findings.

In our study, post-PCNL patients had consistently better clearance across almost all subgroups, particularly for smaller stones (<1.1 cm), middle and lower calyceal locations, and in those with lower BMI. The only exceptions were upper calyceal and PUJ stones, where clearance rates were similar between groups.

Several factors may explain the superior response of post-PCNL residual stones. These stones are often partially fragmented during the initial procedure, weakening their structure and making them more susceptible to shock wave disintegration [17]. Their altered surface morphology and reduced size enhance energy absorption and fragmentation efficiency [18]. Small fragments are also less likely to be embedded in papillae and more easily mobilized into the collecting system for clearance [7].

In contrast, primary stones may be harder, denser, and more firmly anchored within the collecting system. Stone density, commonly measured by Hounsfield units (HU) on CT, is a well-known predictor of ESWL success [19,20]. In our study, we excluded primary stones with HU >1000 because of their consistently poor fragmentation rates. However, in the post-PCNL group, patients were included even if their pre-PCNL CT showed stones with HU >1000. Interestingly, these residual fragments still demonstrated favorable clearance with ESWL. A likely explanation is that the mechanical energy applied during PCNL partially disrupted the crystalline structure of the stones, weakening intramolecular bonds and rendering them more vulnerable to subsequent shock wave disintegration.

The stone location also played a role. Lower calyceal stones were associated with reduced clearance rates in both groups, consistent with prior literature attributing this to unfavorable calyceal anatomy [6,7,21]. Nevertheless, post-PCNL patients achieved higher clearance in this location compared to those with primary stones, possibly reflecting altered post-procedural anatomy that facilitated fragment mobility.

Patient factors such as age, gender, and BMI had minimal statistical influence, although younger patients and those with BMI <25 kg/m² had slightly higher clearance rates. As shown previously, obesity may impair ESWL efficacy due to increased skin-to-stone distance and attenuation of shock wave energy [22].

Clinical implications

The management of post-PCNL residual stones is clinically important, as even small fragments can cause recurrence, obstruction, infection, and pain [9,10]. Repeat PCNL is often considered but is invasive, requires general anesthesia, and carries higher morbidity [11]. Our findings support ESWL as a safe and effective alternative for small post-PCNL residual stones. Flexible ureteroscopy (FURS) is another minimally invasive option with high clearance rates, but it requires anesthesia, higher costs, and specialized expertise. In contrast, ESWL can be performed on an outpatient basis and may be the more practical first-line option in resource-limited settings.

Strengths and limitations

Strengths of this study include its prospective design, equal group sizes, and standardized treatment and follow-up protocols. However, there are limitations. We relied on ultrasonography and KUB X-ray for follow-up, which are less sensitive than non-contrast CT in detecting small residual fragments. The follow-up duration was limited to eight weeks, preventing long-term recurrence analysis. We also did not routinely perform stone composition analysis, which could have provided insight into the differential ESWL response. Additionally, although patients with HU >1000 were excluded from the primary stone group, post-PCNL patients were included regardless of density. Outcomes were not stratified by HU within the post-PCNL group, which limits the interpretation of ESWL efficacy in high-density fragments.

Future directions

Future research should include larger multicenter trials, CT-based follow-up to improve accuracy, long-term recurrence assessment, and cost-effectiveness comparisons between ESWL, flexible ureteroscopy, and repeat PCNL. Incorporating patient-reported outcomes such as recovery time, pain scores, and quality of life would further guide individualized treatment decisions.

## Conclusions

This study demonstrates that ESWL is significantly more effective in treating residual stones after PCNL than in primary stones of comparable size (5-15 mm). Patients with post-PCNL stones were 1.6 times more likely to achieve stone-free status (RR 1.60, 95% CI 1.24-2.07), with consistently higher clearance across most subgroups, except for upper calyceal and PUJ stones, where outcomes were similar. This advantage likely reflects structural weakening of stones during PCNL, which enhances their susceptibility to shock wave fragmentation.

ESWL was safe in our cohort, with no major complications and only minor, self-limiting adverse effects. Compared with repeat PCNL, it avoids anesthesia and surgical risks, and compared with flexible ureteroscopy, it remains more widely accessible in resource-limited settings. In clinical practice, ESWL should be regarded as a first-line option for managing small residual fragments following PCNL, offering a safe, effective, and practical alternative to repeat percutaneous intervention.
